# Preface to ‘Newton, *Principia*, Newton Geneva Edition (17^th^–19^th^) and modern Newtonian mechanics: heritage, past & present’

**DOI:** 10.1098/rsta.2025.0092

**Published:** 2025-07-17

**Authors:** Raffaele Pisano

**Affiliations:** ^1^ HOPAST at IEMN, Department of Physics, University of Lille-CNRS, Lille, France

**Keywords:** Newton’s mechanics, Newton *Principia Geneva Edition*, Jacquier, Le Seur, Calandrini, Footnotes, 1739-1742, 1822

## 1. Newton Geneva Edition research project

Raffaele Pisano and Paolo Bussotti's *Newton Principia Geneva Edition International Project* was born in 2010 during a Mathematical and Physical Workshop at the Italian Naval Academy in Livorno, Italy. In 2014, a book project proposal based on the project was accepted by Oxford University Press (5 vols., expected in 2030) and numerous events and publications on the Newton Geneva Edition have since followed. This project aims to analyse the relationship of the history of physics/mathematics and scientific thought with the analysis and the translation of the Geneva Edition (edition published in Glasgow, 1822; 4 vols.) as a reference frame. It also proposes a new perception and effect of Newton’s theory and how they are communicated.

This issue of Philosophical Transactions of the Royal Society A, *Newton, Principia, Newton Geneva Edition (17th−19th) and Modern Newtonian Mechanics: Heritage, Past & Present*, is linked to a recent international two-day symposium that in September 2023, in collaboration with Paolo Bussotti (University of Udine, Italy), I organized at the University of Oxford, UK, to celebrate 200 years since Newton’s *Philosophiæ Naturalis Principia Mathematica Geneva Edition* ([1739−1742] 1822; hereafter abbreviated to GE), and to present our most significant recent findings on the subject.^1^ The scientific and historical programme included an examination of the Geneva Edition of Newton’s *Principia*. A key aspect of the discussions focused on the historical and scientific characteristics of this edition, including a history of physics and mathematics, as well as its typographical and fundamental attributes.

— The GE was published for the first time in Geneva between 1739 and 1742. A second edition in the same city appeared in 1760. In 1822, a third edition was published in Glasgow. This 1822 edition is a reprint of the third and last edition of the *Principia* (1726; first edition: 1686−1687; second edition: 1713) and several errors were fixed with respect to the original *Principia* Geneva editions (1739−1742).
— The GE editors were two French mathematicians, Thomas Le Seur (1703−1770) and François Jacquier (1711−1788). They worked with Jesuits but belonged to the order of *minim friars*. A third soul, the Swiss Jean-Louis Calandrini (1703−1758)—the unofficial editor—provided essential support to the edition with his physical notes/treaties on Newton’s mechanics and mathematical/geometrical original explanations. The main French editors conserved and explained Newton’s geometrical methods, which were progressively abandoned both in England and on the continent. Therefore, the GE was/is an important hub for European physics–mathematics.
— The 1822 Newton Geneva Edition is composed of 3 volumes. The third is divided into two tomes. Newton’s propositions are detailed, annotated and commented on via footnotes: mathematical and physical aspects, geometrical proofs, methodology, discoveries and advancements after Newton’s works are astutely reported. The footnote commentaries (4 vols.) are longer than Newton’s texts of the *Principia* (3 vols.).
— The GE, enriched by the crucial footnotes and commentaries of the editors, is still only available in Latin language.
— With Oxford University Press, we shall publish the first and complete commented translation from Latin to English of this huge editorial and research enterprise. We shall also add our historical and scientific comments to all footnotes of the GE, side by side, including an additional detailed large introductory work (1 vol.) in which the themes here outlined will be developed in detail. We do not translate Newton’s text; we shall translate the whole apparatus of the footnotes. We refer to the edition printed in 1822 because it offers improvements over earlier versions. Our commentary also looks at primary sources pertinent to the Geneva Edition and its very manuscript format, as well as preceding commentaries on Newton’s *Principia*.

## 2. The essays of Newton Geneva Edition theme issue into three parts

The original research essays of this theme issue look at the main features of the GE associated with the development of Bussotti and Pisano’s research on the subject. It also analyses the scientific and editorial roles played by Le Seur, Jacquier and Calandrini, the scientific nature of the footnotes as well as the GE, which can be seen as the centre from which scientific topics, such as mechanics and its methodology, as well as other geometrical and mathematical subjects connected to the contextual history of science, stem. The original research contributions of the theme issue are organized in the following three parts:

— *Part One*. An introductory research essay on the state of the art of the GE encompasses the legacy of Newton’s mechanics and the popularization of his ideas, particularly as they relate to the different editions of his works. This essay also presents the structure of the GE research and provides justification for its pivotal role in the scientific discourse and policy of science until the first half of the nineteenth century.
— *Part Two*. Scientific and historical research on: the Newton GE in the scientific networks of Cramer and Calandrini; an analysis of the problem of isochronism, Propositions XLVIII–LIII in GE; Newton and compound interest; Newton’s astronomy in GE, Book Three—Propositions XIII–XIV; a new analysis of the three-body problem, Proposition LXVI–Theorem XXVI.
— *Part Three*. Scientific research on Newton’s *Principia* and Newtonian mechanics in the networks of the advanced/modern physical, mathematical and engineering sciences and related disciplines: the origin of quantum mechanical statistics and human language; the separability problem in quantum mechanics and human language; higher-order dynamical equations; Newtonian mechanics and mechatronics; Newtonian analogies in spherical spacetimes, gravitoelectromagnetism and Hawking mass; non-locality of energy in general relativity via Newtonian explanation; Newtonian cosmology and the modern theoretical cosmology; modern disciplines with a Newtonian origin.

This theme issue is an accessible avenue to understanding the richness and the scientific–historical fascination of Newton’s *Philosophiæ Naturalis Principia Mathematica Geneva Edition*, as well as offering much-needed insights into the relationship between physics and mathematics, mechanics and fundamentals. It appeals to scientists and historians of physical and mathematical sciences.

## Data Availability

This article has no additional data.

## Footnotes

^1^

https://newtongenevaedition.sciencesconf.org

